# Sex & vision I: Spatio-temporal resolution

**DOI:** 10.1186/2042-6410-3-20

**Published:** 2012-09-04

**Authors:** Israel Abramov, James Gordon, Olga Feldman, Alla Chavarga

**Affiliations:** 1 Psychology, Brooklyn College, City University of New York, Brooklyn, NY, 11210, USA; 2 Cognition, Brain, and Behavior, The Graduate Center, City University of New York, New York, NY, 10016, USA; 3 Psychology, Hunter College, City University of New York, New York, NY, 10065, USA; 4 Biopsychology and Behavioral Neuroscience, The Graduate Center, City University of New York, New York, NY, 10016, USA; 5 Center for Neural Science, New York University, New York, NY, 10003, USA

## Abstract

**Background:**

Cerebral cortex has a very large number of testosterone receptors, which could be a basis for sex differences in sensory functions. For example, audition has clear sex differences, which are related to serum testosterone levels. Of all major sensory systems only vision has not been examined for sex differences, which is surprising because occipital lobe (primary visual projection area) may have the highest density of testosterone receptors in the cortex. We have examined a basic visual function: spatial and temporal pattern resolution and acuity.

**Methods:**

We tested large groups of young adults with normal vision. They were screened with a battery of standard tests that examined acuity, color vision, and stereopsis. We sampled the visual system’s contrast-sensitivity function (CSF) across the entire spatio-temporal space: 6 spatial frequencies at each of 5 temporal rates. Stimuli were gratings with sinusoidal luminance profiles generated on a special-purpose computer screen; their contrast was also sinusoidally modulated in time. We measured threshold contrasts using a criterion-free (forced-choice), adaptive psychophysical method (QUEST algorithm). Also, each individual’s acuity limit was estimated by fitting his or her data with a model and extrapolating to find the spatial frequency corresponding to 100% contrast.

**Results:**

At a very low temporal rate, the spatial CSF was the canonical inverted-U; but for higher temporal rates, the maxima of the spatial CSFs shifted: Observers lost sensitivity at high spatial frequencies and gained sensitivity at low frequencies; also, all the maxima of the CSFs shifted by about the same amount in spatial frequency. Main effect: there was a significant (ANOVA) sex difference. Across the entire spatio-temporal domain, males were more sensitive, especially at higher spatial frequencies; similarly males had significantly better acuity at all temporal rates.

**Conclusion:**

As with other sensory systems, there are marked sex differences in vision. The CSFs we measure are largely determined by inputs from specific sets of thalamic neurons to individual neurons in primary visual cortex. This convergence from thalamus to cortex is guided by cortex during embryogenesis. We suggest that testosterone plays a major role, leading to different connectivities in males and in females. But, for whatever reasons, we find that males have significantly greater sensitivity for fine detail and for rapidly moving stimuli. One interpretation is that this is consistent with sex roles in hunter-gatherer societies.

## Background

Vision begins with the image focused on the retina at the back of the eye. One way of examining the first stage of processing of that image is to measure ability to detect elementary changes in light and dark across the image; because the image probably also varies with time, we also measure the effects of different rates of change, to approximate changes that might occur in real-world viewing. We are dealing here with the effects of sex on these elementary visual sensations.

At least one sex-effect is well-known. Color vision depends on three types of cone photoreceptors in the retina: some are more sensitive to the longer wavelengths of light (L-cones), some to the middle wavelengths (M-cones), and some to the shorter wavelengths (S-cones). The genes coding for two of these cone photoreceptors (L- and M-cones) are carried on the X-chromosome and any malformation of either gene in a female is necessarily expressed in the phenotype of a male offspring who inherits that gene. However, it seems to be tacitly assumed that there are no other major sex effects on visual capacities. (But see companion paper [1], for sex effects on color appearance.) Of course, major clinically oriented surveys that deal mostly with acuity and optical issues test large samples from a population and examine many variables, including sex. For example: this was done in the Hispanic Health and Nutrition Examination Survey of Hispanic and non-Hispanic populations – in fact this study found no significant sex-related acuity differences in children and adolescents [2]. However, other studies of visual acuity have reported significant sex differences in adults [3,4].

Despite mandates of major granting agencies to include, where possible, equal numbers of males and females in studies, little attention is paid to sex differences. For example, we examined all papers published in 2010 in a major journal, Vision Research. We tabulated all studies that used psychophysical or physiological measures to test humans’ basic capacities, such as spatial and temporal resolution, stereopsis, motion detection, and so on. We identified 410 such studies, of which only 96 (23.4%) broke down their samples into males and females. Also, at least in this sample of publications, only 4 studies (less than 1%) used sex as a comparison variable.

The lack of attention to sex differences in vision is surprising given the considerable body of work comparing male and female visually based perceptual and cognitive abilities: for example, females are said to make many more fine distinctions among colored objects [5,6]. But if the initial information is processed differently according to sex, at least some of the “higher” differences might be due to differences in the results of this processing. Despite this possibility, little attention has been lavished on sex differences in basic visual sensations that are determined at “early” stages of the eye and brain.

Sex differences have been examined in other sensory systems: all showed clear sex differences. In the auditory system, there were differences in electrical responses of the brain (auditory evoked potentials); also ears of females were more likely to produce spontaneous emissions of sounds from the ear (otoacoustic emissions), and in those females, hearing sensitivity was better than in males. All these differences could be related to the masculinizing effects of androgens [7-9]. Similarly, for the olfactory system, it seems clear that, in most cases, females had better sensitivity, and discriminated and categorized odors better than males [10,11]. The general conclusion is that for audition and olfaction, as well as taste and somato-sensory sensitivity, females have greater sensitivity than males [12].

The substrate for these sex differences may be linked directly to gonadal steroid hormones: in rhesus monkeys, large numbers of androgen receptors are found on neurons throughout the cerebral cortex, including visual cortex. This androgen binding “may have considerable impact on cortical functioning in primates at postnatal as well as prenatal ages” [13]. There are similar findings for rats, in whom males have more androgen receptors than females, and these are especially plentiful in primary visual cortex [14]. A recent review has reiterated these findings and concluded that in both humans and rats the largest concentration of androgen receptors in the forebrain is in the cerebral cortex and not the hypothalamic and limbic areas associated with reproduction [15]: these findings might be general across mammals. All the above authors strongly emphasize that the distribution of androgen binding receptors may have considerable impact on cortical development and maturation of visual functioning.

Furthermore, in rats, androgens, but not estrogen, directly modify development of the visual cortex. Androgens reduce the early post-natal cell-death (apoptosis) of the visual cortex; as a consequence males have 20% more neurons in the visual cortex [16,17]. This organizational effect is androgen-specific: early exposure of female rats to androgens (implanted capsules of dihydrotestosterone) led to these effects; early exposure to estrogen (implanted capsules of estradiol) did not inhibit post-natal cell-death [16].

Factors other than gonadal receptors may also be involved [18]. Theoretically, females might have a double “dose” of sex-related genes. To compensate for this, one of each pair of X-chromosomes is silenced [19]. Many humans have multiple L and M genes – we are polymorphic for these genes [20,21]. As a consequence, different retinal areas might express different alleles, affecting the responses of these areas and the brain sites associated with different retinal areas. Moreover, the X-chromosome may have a loading of “male-benefit” genes: thus, any recessive alleles must, of necessity, be expressed in a male [18]. Furthermore, some of the sex effects we report here could be either organizational or activational and could depend on estrogen rather than testosterone; they could even be due to other sex-related genes [22].

In short, it seems highly unlikely that vision will differ from other sensory modalities that show sex differences in function. It seems parsimonious to assume that the plethora of androgen receptors and androgen effects in primary visual cortex exert a measurable and important impact on visual sensory capacities.

We study differences between males and females in basic visual functions. The work we report here is not directly hypothesis-driven – it is exploratory. Our weak hypothesis is based on the fact that males have higher levels of androgens and that there are large numbers of testosterone receptors in visual cortex: therefore we anticipate sex differences. We cannot formulate a strict hypothesis because we cannot manipulate testosterone levels, nor have we measured them. This problem is endemic to most studies of sex differences in adult populations and is exacerbated by the possibility that some of the differences might not even be androgen driven.

We have developed a Battery of Visual Tests designed to study different visual sensory capacities, each with state-of-art precision. Tests include: (i) ability to resolve/detect patterns that vary in space (from coarse to fine detail ) and time (from very fast to very slow rates of change) -- these measures determine the spatio-temporal contrast-sensitivity function; (ii) detecting a small offset between two lines (vernier acuity); (iii) motion detection; (iv) binocular depth perception (stereopsis); and (v) color vision -- especially color appearance (see [1]). One of the reasons for choosing these specific tests is that each emphasizes a different level and locus in the central nervous system. Specifically, the contrast-sensitivity function probably depends on the responses of neurons in primary visual cortex (discussed below); motion detection probably depends on responses of neurons in area MT of the temporal lobe; and some aspects of color appearance depend on an intact infero-temporal cortex (e.g., [23,24]).

In this paper we report on sex differences in spatio-temporal contrast-sensitivity. Spatial contrast-sensitivity (S-CSF) describes the visual system’s ability to perceive changes in brightness across space, as in reading a letter on an eye chart, or recognizing a face. The details of our procedures are given below.

The protocol for each measure in our Battery of Visual Tests is rigidly controlled. A consequence is that the thresholds we record from individuals now are precisely comparable to those recorded from individuals tested years ago. To minimize run-time errors, either the apparatus as a whole or the run-time stimulus sequence and data collection are computer-controlled. When these thresholds are measured, a participant adopts an internal criterion for reporting when a stimulus is visible; unfortunately these criteria vary across time within and among participants. Where possible, therefore, we use criterion-free psychophysical procedures: the stimulus is presented in one of two alternative positions or time intervals; after each presentation participants must choose where the stimulus occurred, regardless of confidence in their choice; stimulus strength is reduced from trial to trial until correct responding reaches chance levels. This “forced-choice” procedure is criterion free. In practice, we use a modified Bayesian approach to vary the stimuli in order to find threshold; trials are continued until the estimates of threshold reach an asymptote at a specified (99%) statistical confidence level (QUEST algorithm; [25]). Also, we attempt to test each participant on the entire battery of tests to examine possible interactions among different capacities – those capacities whose thresholds are correlated presumably share a neuronal substrate.

We have amassed large databases from participants who participated in most or all of the Battery’s tests. We now have sufficient data to examine possible sex effects. Even if effect magnitudes are small or subtle, they cannot be ignored because they point to our hypothesized developmental and maturational effects of gonadal hormones on very basic sensory functions.

The following describes how, in the laboratory, we measure the S-CSF: we use grating patterns (alternating light and dark bars); the gratings vary from broad, coarsely spaced bars to fine, closely spaced bars. On each trial the grating pattern is either horizontal or vertical and the participant must choose between these alternative orientations. The spacing of the bars is measured as the number of light–dark cycles within one degree of visual space (cy/deg). The change from light to dark bars follows a sinusoidal profile in which intensity is varied symmetrically above and below a mean gray level; the difference between maximal and minimal intensities of the pattern is reduced until the pattern is no longer detectably different from a uniform gray – that is, the grating’s contrast is reduced until the pattern is at threshold.

The gratings had sinusoidal profiles because any image can be synthesized from (is equivalent to) a specific set of sinusoids of different frequencies, amplitudes, and phases (Fourier’s theorem). Limiting measures of spatial resolution only to the finest detail that can be perceived (acuity) is akin to testing auditory capacities using only the top end of the piano keyboard. High spatial frequencies are indeed important for discriminating among images that are similar to each other. However, sensitivity to the lower spatial frequencies greatly influences our ability to recognize and categorize parts of the image that refer to the diverse objects on the visual field [26-28].

Similarly, temporal contrast-sensitivity (T-CSF) refers to the visual system’s ability to perceive changes in brightness over time, as when the stimulus flickers, or the retinal image slips across the retina due to object and/or viewer motion. However, S- and T-CSFs are strictly non-separable functions: the precise function that is obtained depends on the values of both sets of parameters – these define an entire three-dimensional sensitivity surface. Kelly, an early advocate of this non-separability [29,30], used gratings of each spatial frequency which moved horizontally at a fixed velocity. This was impractical in our situation so we chose to measure the entire spatio-temporal (ST-CSF) surface using a series of spatial gratings, each of whose contrasts was modulated at each of a fixed series of temporal rates. The time-profile of the modulation was sinusoidal. The units for these temporal rates are variation-cycles/s (Hz).

## Methods

### Participants

All participants were volunteers, drawn from undergraduate and graduate students, and faculty at Brooklyn College, together with some high school students. The demographics of student participants parallel the demographics of the student body at Brooklyn College.

We wanted to make sure that our participants had “normal” vision across most of the basic visual capacities – we could not assume, a priori, that there are no interactions and that some other aspect of vision had no impact on the visual capacity we describe in this paper. We therefore screened all participants using standard measures of the visual capacities that seemed relevant.

We deal here with sensitivity to patterns that vary across the visual field. We started by ruling out purely optical factors – how well is the image focused on the retina? Thus, all participants had acuities of, or were corrected to, at least 20/20 and were tested with their best optical correction; acuity was measured using standard letter charts (Snellen optotypes).

Individuals with anomalous color vision are not included in this paper because they can have better acuities (including spatial resolution in general) than the rest of the population [31-33]. All our participants had normal color vision. We first “screened out” all with clear anomalies as detected with the familiar plates of figures composed of dots of different colors (Dvorine pseudo-isochromatic plates; Harcourt, Brace & World). This was followed by tests to assess more accurately the quality and precision of their color vision (Farnsworth Dichotomous Test for Color Blindness, Panel d-15, Psychological Corp. and Lanthony’s Desaturated 15 Hue Test, Luneau Ophtalmologie, Paris).

Binocular depth perception (stereopsis) is a very fine measure of spatial resolution – in depth, rather than across the frontal visual field. Because this might be correlated with the resolution measures reported here, we assessed stereopsis using standard clinical tests (Titmus Fly Book Test and Randot Stereo Acuity Test, Stereo Optical Co., Chicago; also, the TNO anaglyphic random-dot plates, Lameris Instrumenten, Holland).

For inclusion in this study, data were drawn only from participants with ages between 16 and 38 years; our database shows that over this range there is little change in CSFs. There were 36 females, aged 16–38 (mean = 23.5) and 16 males, aged 16–37 (mean = 24.3). These numbers of participants are appreciably larger than those in the vast majority of detailed psychophysical studies of vision. Furthermore, each of our participants had to complete our entire Battery of tests of vision; this entailed coming to the laboratory for fifteen or more sessions of approximately one hour. The smaller number of males reflects the College’s demographics; also, we excluded everyone with anomalous color vision; as expected, these were males.

The study was approved by the Institutional Review Board of Brooklyn College, where all the studies were conducted. All participants were volunteers and gave informed consent to participate in this study. The experiments were conducted in accordance with the principles embodied in the Declaration of Helsinki (Code of Ethics of the World Medical Association).

### Stimuli

We measured the spatial and temporal resolution of the visual system to monochrome targets that varied in contrast, while keeping constant the mean luminance across the entire pattern. Stimuli were sinusoidal gratings whose Michelson contrasts (luminances: ((max-min)/(max + min)) varied from near zero to near 100%. There were 6 spatial frequencies (0.6, 1, 2, 5, 12, and 24.4 cy/deg) and 5 temporal frequencies (1, 4, 8, 15, 24 Hz); temporal modulation was counter-phase (light bars changing to dark, and dark bars changing to light) with a sinusoidal temporal profile. Each spatial frequency was modulated at each of the temporal frequencies, giving a total of 30 different stimuli to define a spatio-temporal surface. There was no static condition (grating stimuli that were not modulated in time); this was done for analytic reasons (logarithmic abscissa). Also, there were practical considerations: eye movements prevent a true static condition; however, at our relatively short presentation duration, the 1-Hz condition approximates a static condition.

### Apparatus

The apparatus was a dedicated computer and interface driving a monitor (Vision Works Graphics Displays). The display was broad-band monochrome (CIE 1931 coordinates, x = 0.443 and y = 0.533) with a mean luminance of 55 cd/m^2^. (Approximately the brightness of a standard TV screen.) The display screen was seen through a circular aperture (3.5^0^) in a white surround screen (13^0^ x13^0^), front-illuminated at approximately 25 cd/m^2^ (CIE 1931 coordinates, x = 0.523, y = 0.417). Calibrations were made with a scanning spectro-photometer (Photo Research, model 704A).

Participants viewed the stimuli binocularly and wore their own corrective lenses, if available, or were provided with the appropriate lenses in a trial-frame. There was no fixation target; rather, participants were told simply to look at the center of the screen. They were seated, in a darkened room, 3.6 m from the display with their heads stabilized with a chin-rest.

### Procedure

Two sessions were needed to measure the entire spatio-temporal sensitivity function. The participant’s task was to identify whether a grating was horizontal or vertical. There was no explicit training session.

In the first session participants viewed each of the 30 stimuli (random order and randomly horizontal or vertical). To establish an approximate contrast threshold for each stimulus, the participant adjusted contrast until the orientation of the stimulus could just be detected. These adjustment data were used to set starting points for obtaining the final thresholds. This was done because it was important that the starting contrasts be approximately the same distances from their final thresholds in order to avoid biases. Then in the same session a random subset of 15 of the 30 (spatial × temporal frequencies) stimuli was used to obtain the final thresholds (see below for details); the remaining stimuli were presented in the second session.

The final thresholds were obtained using a two-alternative, forced-choice procedure – on each trial, the participant had to choose whether the grating was horizontal or vertical. On any given trial, the contrast of the grating depended on the participant’s performance on the preceding series of contrasts. The specific algorithm used a Bayesian estimate of threshold (QUEST see [25]). A QUEST stimulus-presentation had fixed duration (2 s). To minimize transients, on any given trial, a stimulus was “ramped” on and off (contrast increased linearly from zero) for 0.5 s and remained at full contrast for 1 s. The 15 stimuli in a session were presented in 3 blocks of 5 interleaved sequences. Trials were “self-paced” – the participant simply started the next trial when ready. Each trial displayed a different stimulus. The forced-choice algorithm was used to avoid participant biases such as response criterion, that is, the level of contrast required by the participant before acknowledging that a pattern was visible [34]. The participant could decide either during or after the observation interval. Between observations, the display screen was uniform at the mean luminance level. Participants entered their responses via a computer keyboard with a plastic cover exposing only the relevant keys.

Feedback was provided for correct answers (the computer beeped) and performance determined the contrast on the next trial on which that grating appeared. Each contrast series was continued until the derived threshold reached the 99% confidence level.

## Results

### Spatial Contrast-Sensitivity Functions (S-CSFs)

**Figure 1:**
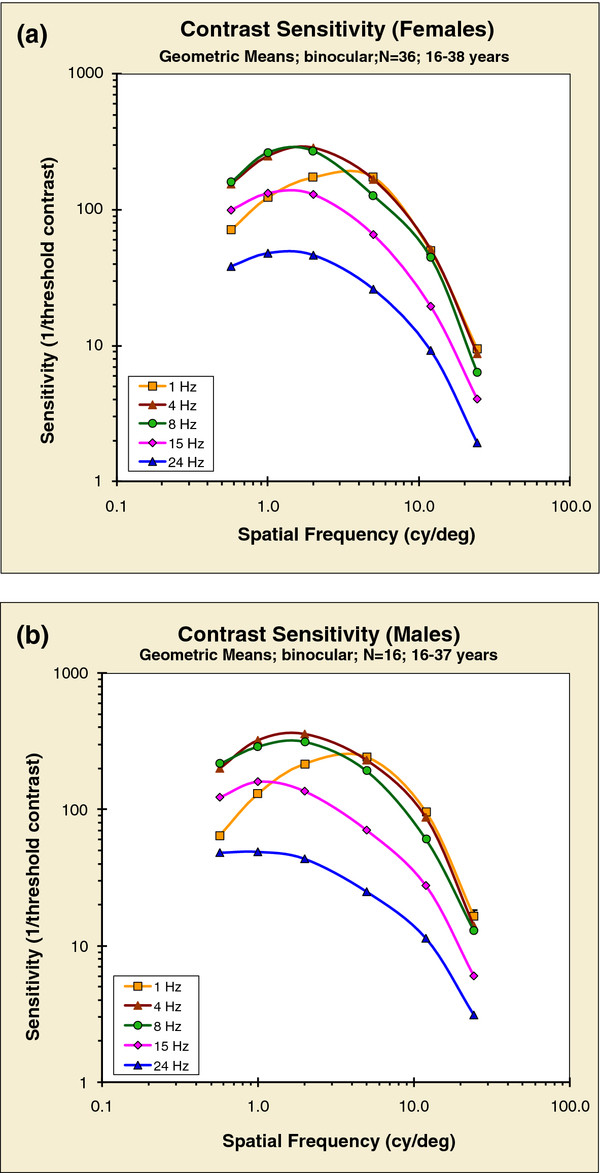
**Binocular spatial contrast-sensitivity functions, parametric in temporal modulation rate (group geometric means); all participants color and stereo normal: (a) Females (N = 36; 16–38 years); (b) Males (N = 16; 16–37 years).** Error bars are not shown because the SEMs are largely encapsulated within the size of each data symbol.

Group mean S-CSFs are shown in Figure 1. The axes are log-log for the following reasons: this follows commonly accepted practice; more cogently, a log abscissa is appropriate because the underlying spatial sensitivity channels have equal log bandwidths [35-38]. Also, a log ordinate is useful because of the huge range of sensitivities. Such an axis suggests that the appropriate average is the geometric mean: we computed both arithmetic and geometric means; for the arithmetic means, group variances were positively correlated with the means (R = 0.83 for males and 0.72 for females). Using geometric means, however, “normalized” the variances so that means and variances were less well correlated (R = 0.35 for males and 0.44 for females).

Figure 1a presents the data from females, and 1b for males, parametric in temporal modulation rate. Each family of curves reiterates the observation that there is no single CSF, rather a spatio-temporal surface [29,30]. The 1-Hz condition (approximating the usual static condition) follows the familiar inverted-U shape. Temporal modulation changes the shape of the S-CSF: for moderate temporal rates (4 and 8 Hz) there is a slight loss of sensitivity at the high spatial frequencies, but a marked increase in sensitivity at low spatial frequencies, while for the higher temporal rates there is an appreciable drop in sensitivity at all spatial frequencies. Finally, for all temporal modulation rates greater than 1-Hz, each spatial CSF, was shifted, in its entirety, towards lower spatial frequencies; each CSF is shifted by approximately the same amount. (Our findings about the change in the shape of the S-CSF with temporal modulation are consistent with earlier reports: [39,40]).

The between-participant variability was very small: Standard Errors of the Mean, both for males and for females, were all less than 0.35 across all thirty stimulus conditions. Figure 1 does not include any error bars because they are largely the same size as the symbols for the means.

An individual’s Spatio-temporal Contrast-Sensitivity (ST-CSF) is a three dimensional surface. We fitted each surface with a mesh that best describes the surface [41]. From this mesh we estimated the spatial frequency of maximum sensitivity for each temporal rate. Thus, for females the group mean of these spatial frequencies, for 1-Hz modulation rate, was 3.42 cy/deg; for all the other S-CSFs, the peak frequencies ranged from 1.72 to 1.91 cy/deg. The same pattern was found for males, except that the shifts were more pronounced: for 1-Hz, mean peak was 3.69 cy/deg, while the peak frequencies for all the other modulation rates ranged from 1.11 to 1.62 cy/deg.

**Figure 2:**
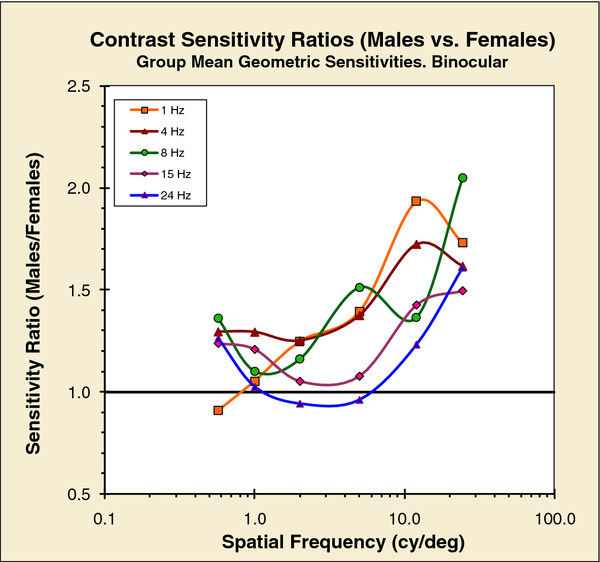
**Group comparisons of binocular spatial contrast-sensitivity functions in Figures**1**a,**1**b: ratios of mean sensitivities for males to females.** Ratios greater than one show that males have higher sensitivity than females.

There are clear sex-related differences in ST-CSFs. While these differences are not immediately apparent from a simple comparison of Figure 1a and 1b, the differences become strikingly apparent when we examine ratios of sensitivities of males to females. Figure 2, shows these ratios for each of the 30 stimuli (6 spatial × 5 temporal), computed from the data in Figure 1. Except for three points close to one, all ratios are greater than 1.0: males have greater sensitivity than females, and this male advantage increases with spatial frequency. (A two-tailed sign-test shows that for males to have greater sensitivity than females for 27 out of 30 stimulus conditions is unlikely, p < 0.0001.) Interestingly, for both sexes, the coincidence of 4 and 8 Hz curves in Figure 1a and 1b reveals that the ST-CSF surface is flat in this vicinity – it has a plateau.

An ANOVA (SPSS Version 11.5; general linear model, repeated measures, mixed design) was run: the factors were sex, spatial frequency, and temporal modulation rate. The effect of sex was significant: F(1, 50) = 4.145, p = 0.047. There was also a significant interaction: sex × temporal rate: F (9, 42) = 5.171, p = 0.004. There were no other significant effects or interactions.

We calculated effect-sizes (Cohen’s d) separately for each of the 30 stimulus conditions; effect sizes ranged from 0.01 to 0.8. The smaller effect-sizes were associated with the lower spatial frequencies, for which sex differences were small. The larger effect-sizes were associated with the higher spatial frequencies, for which the sex differences were larger. The reason for this trend is clear in Figure 2, which shows the ratio of male-to-female sensitivities (range 1.2 to 2): for example, at 0.6 cy/deg median Cohen’s d = 0.38 (range, 0.15 to 0.47); at 24 cy/deg median Cohen’s d = 0.54 (range, 0.36 to 0.8).

**Figure 3:**
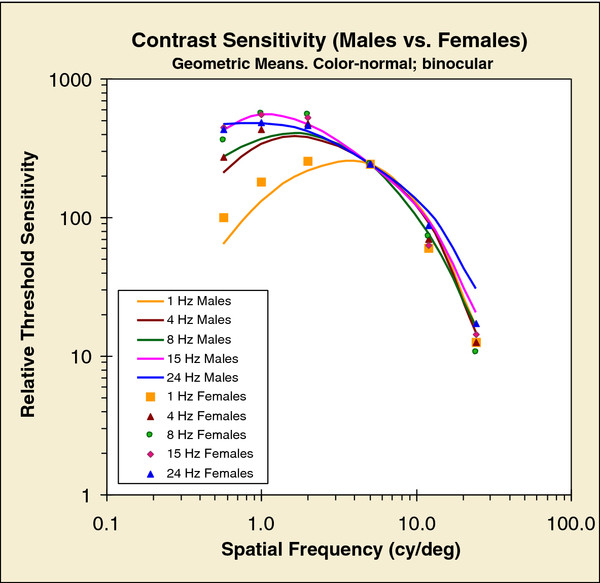
**Contrast-sensitivity functions of males compared to those of females.** Mean functions for males scaled to coincide at 5 cy/deg for 1 Hz temporal modulation. Mean functions for females scaled separately to coincide at same rate; scaled female functions then scaled, as a group, to match scaled male functions. Females represented by data points, males by lines.

We examined whether the shapes of the S-CSFs differ between males and females at each temporal rate; note that we are considering the shape of each function and not absolute sensitivity. To do this, we first found the scale factors, separately for males and females, needed to pin together the curves in Figure 1 at one chosen spatial frequency. We chose to do this at 5 cy/deg for 1-Hz modulation rate -- 5 cy/deg splits our range of spatial frequencies; it is also the point closest to the peak of the canonical “static” CSF. We then found the scale factor required to shift the pinned curves for females to the pinned curves for males at 5 cy/deg; at that point, mean data for females had to be multiplied by 1.67 to match male data, showing that overall males had greater sensitivities. Figure 3 shows the results of this rescaling: the data points are the rescaled female S-CSFs, while the lines are for males. Although the shapes of the S-CSFs at each modulation rate are very similar for males and females, there is a consistent trend: females are relatively more sensitive at lower spatial frequencies and males are relatively more sensitive at higher spatial frequencies. Finally, this figure re-iterates a point made earlier: any modulation rate greater than 1-Hz shifts all the curves to lower spatial frequencies and all these curves have much the same shape.

Briefly, in Figure 2, the ratio curves do not fall on top of each other, which means that the shapes of the CSFs, for different rates of temporal modulation, change differently for males and females. Figure 3 shows the shape changes in detail: for all temporal modulations less than 1-Hz, all the S-CSFs have approximately the same shape. But, overall, males are more sensitive than females.

### Acuity

The most common measure of spatial resolution is the acuity limit – the finest detail that can just be resolved. But, there is a problem with the usual tests, such as optotypes (letter charts) or gratings printed on “acuity cards:” they vary in fixed steps; thus, spatial frequencies estimated from size of elements can only approximate the actual limit. Although we did not measure directly spatial frequencies for acuity, that limit can be estimated from our S-CSFs. Each participant’s S-CSF, parametric in temporal rate, was fitted with a model function: a double-exponential curve that describes nicely the shape of a spatial CSF [42]. The high frequency (right limb) of each fitted curve (best fitting model, by least-squares criterion), was then extrapolated to find the spatial frequency corresponding to a sensitivity of 1.0 (i.e., 100% contrast); at that frequency the width of half a cycle of the grating corresponds to the acuity limit from standard tests.

**Figure 4:**
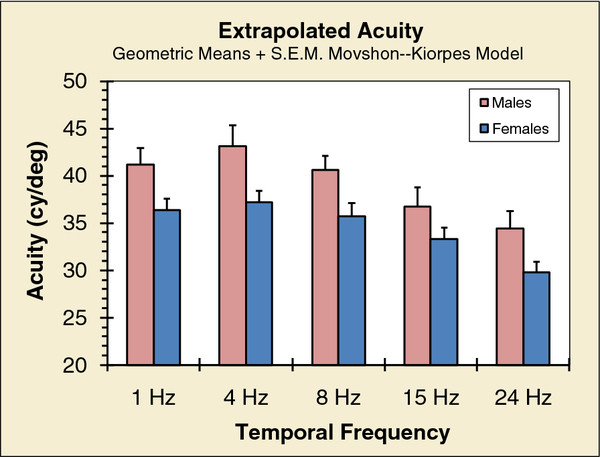
**Limiting acuities of males and females estimated from contrast-sensitivity functions.** Each participant’s function for each of the temporal rates was fitted with a model of these functions [42]. The model functions were extrapolated to find the spatial frequency corresponding to 100% contrast. (Width of half a cycle at that frequency is the acuity limit in units of degrees.) Individuals’ estimated acuities were averaged to obtain group means.

Group geometric-mean acuities were calculated separately for males and females; the results are shown in Figure 4. As expected, acuities fall systematically as temporal rate is increased. However, males had appreciably better acuities at every temporal modulation rate. An ANOVA confirmed this sex-related difference in acuity: F (1, 50) = 6.653, p = 0.013. There were no other significant effects.

## Discussion

### ST-CSF

The CSF can be used to predict an individual’s ability to detect and recognize or categorize targets: sensitivity to high spatial frequencies is directly related to target detection and fine discriminations; but, sensitivity to lower spatial frequencies may be the basis for much of our ability to recognize and categorize objects [26-28]. Object perception involves both holistic (Gestalt-like) and analytic (bottom-up) processes (see [43]); but regardless of one’s preference for one approach or the other, the visual input to both must be filtered through the ST-CSF.

Figure 2 shows that females and males have similar sensitivities at low spatial frequencies; but with increasing spatial frequency, males’ higher sensitivity becomes more and more apparent. (An earlier study that used a much more restricted range of spatial and temporal frequencies, found a similar pattern of male–female differences [44]) The higher sensitivity of males at middle and high spatial frequencies may be common to mammals: the same pattern of sensitivities has been found, using behavioral techniques, in hooded rats [45].

The ST-CSF is probably determined at an early cortical level, whereas object recognition is a higher cortical function. We assert that primary visual cortex (V1) is the major locus for the ST-CSF: most of its neurons are narrowly tuned to specific stimulus sizes and orientations. In fact their receptive fields are best described as responding to a narrow band of spatial frequencies [46,47], and these receptive fields are orientation specific, as is the ST-CSF – an adapting grating is maximally effective when its orientation is the same as that of the test gratings [35]. Furthermore, an adapting grating is effective when it is presented to one eye and the test grating to the other [35] – V1 is the first level at which the neurons are binocular, with matching receptive fields in each eye. But the detection capabilities of V1 neurons that we have just described should be tempered: the spatial tuning of a V1 neuron’s responses changes dynamically according to the prevailing stimulus environment [48,49].

We can only speculate why there is the sex difference that we report here. Are there plausible differences in cortical receptive fields that might account for the difference? One possibility is that females' receptive fields are slightly larger than those of males. Or, males might have greater intra-cortical inhibitory feedback that might increase the degree to which their receptive fields are tuned to finer patterns. Additionally, human males, like rats, may also have 20% more neurons in their visual cortex [16,17]; this alone might increase sampling of the visual image and increase signal-to-noise ratios.

Finally, our data show that any appreciable temporal variation in the stimulus (e.g., 4 Hz and greater modulations) shifts the ST-CSF towards lower spatial frequencies – there is a drop in sensitivity at high spatial frequencies and a considerable improvement in sensitivity at lower spatial frequencies (see Figure 3). Furthermore, all the CSFs shift by almost the same amount, and this shift is not very different between males and females. (There is, of course an absolute difference between the sexes, in that males are generally more sensitive.)

Thus, the canonical static CSF (approximated by our 1-Hz condition) may not be the relevant one in most circumstances – the most relevant may be the shape we obtain when gratings are temporally modulated (see Figure 3). The more usual real-world condition is that the retinal image is far from stationary – either the eye or the entire body moves, or objects in the environment move. In fact it may be ecologically useful that the curves shift in this way: object categorization depends on low spatial frequencies; it is probably more useful to recognize that a moving object is a cheetah without necessarily identifying which cheetah based on fine details of number of spots. Incidentally, even when the world is static and the observer is fixating as steadily as possible, there is still an appreciable temporal modulation of the retinal image: under best fixation conditions, when standing upright without any restraints, there is a drift away from the fixation point of approximately 0.7 deg/s [50]. This implies a 7-Hz modulation rate for a grating (or component of an image) of 10 cy/deg.

There is some evidence from psychophysics with infants that some of the sex effects may have a maturational component that has been speculatively linked to early testosterone surges in males [51].

### Acuity

We find that male acuities exceed those of females both for essentially static and temporally modulated stimuli. Similarly, it has been reported, based on large samples and measured with static and moving stimuli, that males had significantly better acuities under all conditions [3,4]; we have found no studies that show the reverse. The standard Snellen acuity limit is taken as 1 arcmin, which is the width of the line making an optotype for 6/6 (20/20) resolution. Our acuity values (except those for females at a temporal modulation of 24 Hz) imply a bar width less than 1 arcmin; furthermore, the difference between geometric means for males and females is significant (see above).

There are two issues about our acuity findings: why are our acuity values better than the standard 1 arcmin, and why are males better than females? For the first issue we will consider the impact of psychophysical measurement techniques. For the second we will consider a range of possible optical factors. Anticipating the discussion, we can state that none of these factors change our findings that males do better than females across the spatio-temporal domain, including acuity.

Possible psychophysical reasons for acuities better than the 1 arcmin standard include: (a) our use of forced-choice psychophysics, and (b) our use of grating stimuli. Grating acuity is often assumed to be superior to optotype acuity [52]. However, there is no clear evidence for the superiority of grating measures when testing normal adults: (i) gratings (specifically, Teller Acuity Cards) may underestimate acuity as measured with standard letter charts (Snellen optotypes) [53]; (ii) others have reported few or no differences between the two acuity measures when stimuli are viewed foveally; however grating acuity is increasingly superior with increasing eccentricity of viewing – apparently Snellen acuity deteriorates more rapidly across the visual field [54]. We are left with the conclusion that our better than standard acuity values are probably due to the psychophysical algorithm we used: stimulus contrasts are changed from trial to trial until we reach a stringent confidence criterion.

### Optics

Optical factors are known to modify the CSF, especially at higher spatial frequencies (e.g. [37,55]). These effects must be ruled out before asserting that the sex differences we report for ST-CSFs are due to neural differences. We consider only those factors for which separate male and female values are available, or can be inferred plausibly (unfortunately, there is a dearth of direct information and many values must be estimated). The factors are: (i) retinal illuminance, (ii) angular subtense of cones, and (iii) densities of the photopigments in the cones. We restrict discussion to these factors because they are the only ones for which there are sex data, or plausible estimates of sex differences.

 (i) Retinal illuminance

This clearly affects the CSF (e.g., [56,57]), especially when it falls below approximately 100 Td (a measure of retinal illuminance). Retinal illuminance depends on both the eye’s light-gathering capacity, described by its f-stop (focal length/pupil diameter), and the luminance of the stimulus. Pupils of males may be slightly larger than those of females by a small but statistically non-significant amount [58,59]. For our male participants we estimate the average pupil size to be 3.1 mm for our stimulus luminance of 55 cd/m^2^ (approximately 400 Td); we base this on published measures of mean pupil size for different stimulus luminances [60]. For females we scale the male value by the ratio of the dark-adapted diameters for individuals aged 16–19 years – by which ages the dark-adapted diameters have reached their asymptote [59]: the resulting estimate is 3.0 mm, and the ratio of male-to-female diameters is 1.03. However f-stop depends not only on pupil diameter; it also depends on focal length (f.l.).

In lieu of direct measures from both sexes, we estimated f.l. in two ways. Firstly, by assuming that the length of the vitreal chamber closely approximates f.l. – strictly, f.l. is the distance between the nodal point of the eye’s optics and the retina. The relevant nodal point for the eye’s optics lies approximately 0.28 mm in front of the rear surface of the lens [61]; adding this extra distance to vitreal length produces only a small percentage change. From a large sample, mean vitreal length for males: 16.36 mm; mean for females: 16.07 mm [62]. The difference is small and not statistically significant (based on a *t*-test that we performed: t = 0.86). The ratio of male-to-female measures is 1.02. The calculated f-stops are: males, 5.3; females 5.4.

Secondly, if the male eye differs only by a scale factor from that of female, the f-stops must be identical. Mean axial lengths of male eyes are consistently longer than those of females: range across all the cited studies was from 23.73 to 25.54 mm; for males, mean length was 24.28 mm, and 23.74 mm for females [63-65]. The percentage change in the ratio of male to female lengths ranged from 1 to 4% across studies; mean of the ratios, male/female, (across studies) was 1.023. This value for the ratio is very similar to that for vitreal length (see above). Given the close similarity of these ratios of measures for pupil, axial length and vitreal length, it is reasonable to conclude that the eyes of the two sexes differ only by a scale factor and therefore f-stops must be the same, approximately 5.4 under our viewing conditions.

Clearly retinal illuminances are almost the same and cannot account for sex-related degradation of the ST-CSF. But even if retinal illuminances are the same, there may still be sex differences due to differences in the abilities of individual receptors to trap photons and resolve spatial differences in stimuli.

 (ii) Angular subtense of cones

The amount of light funneled into a single cone depends on the area of the cone’s inner segment through which light must pass to reach the outer segment that contains the photopigment. Specifically, this depends on the angle subtended at the nodal point by the diameter of the base of the cone’s inner segment. In the very central retina the cones are as closely packed as possible (hexagonally close-packed) [66,67]. Thus, cone-spacing is effectively the same as cone diameter. This measure defines the retinal mosaic that samples the visual image, which limits acuity [68]. Direct studies that measured cone spacing and acuity in the same participants show that acuity is better in those who have finer receptor mosaics (i.e., smaller cone diameters) [69,70]. Because, to a first approximation, male and female eyes are very similar in focal length, any differences in cone diameter should affect spatial resolution. The diameter of the inner segment is approximately 2 μm [71-73]; for this diameter, the subtense, using the above estimates of focal length, is approximately 0.5 arcmin. This cone diameter would permit detection of gratings of 60 cy/deg, which is better than the 20/20 standard of 1 arcmin: at 60 cy/deg a single cycle has a width of 1 arcmin; the width of a dark bar is, therefore, 0.5 arcmin. However cone diameter depends crucially on precisely which cones are measured: in the very center of the fovea cones can be as narrow as 1 μm, implying even better resolution. Clearly, a major limiting factor for acuity is diameter of the cone’s inner segment. However, we have no evidence that females’ cones are larger than those of males, which might have accounted for our reliable difference in acuity.

Apertures of the cones, however, are not the only relevant factor: receptor pooling must also be considered. It is generally accepted that the diameters of the centers of neuronal receptive fields determine local retinal spatial resolution. Based on the above cone diameters, the centers of the relevant neurons would have to be driven by single cones, which is the case for the midget ganglion cells that predominate in the central retina [74]. To account for sex-related differences in acuity, females would have to have many fewer midget-system cells.

 (iii) Cone photopigment density

Once a photon is funneled into a cone, the probability that it will stimulate the cone depends on the total number of photopigment molecules in that receptor; this number is determined by the length of the pigment-containing outer segment and the density of molecules – i.e., absorbance. At least for density, there are no sex-related differences: transverse absorbances (i.e., perpendicular to long axes of the cones) are the same for both L- and M-cones in humans [75]. Thus, to account for acuity differences, the lengths of the outer segments would have to be quite different for males and females; the ratios (M/F) of acuities for each temporal rate (see Figure 4) range from 1.10 to 1.16 with a mean of 1.14. Unfortunately, we do not have any relevant anatomical data.

In conclusion, we find no obvious optical explanation for the sex differences we find (Figure 2). Sex differences in eye size, f-stop, and focal length are approximately 1%. Yet acuity of males is some 5 cy/deg higher than that of females (Figure 4), which is approximately a 10% difference. Furthermore, we cannot find any explanation of the acuity differences based on properties of the cones; the diameters of the cones’ inner segments (cone entrance pupil) and the lengths of the photopigment-containing outer segments do not seem to be different – the differences would have to be large to account for 10% differences in acuity.

### Receptive fields and sex-differences

We emphasize that the sex-differences we find are important, especially for understanding the embryogensesis and maturation of the primary visual centers. To provide V1 neurons with elongated and oriented receptive fields, the axonal inputs from the lateral geniculate nucleus of the thalamus (LGN) must be quite specific. Receptive fields of LGN neurons are circular with opposed responses from center and surround (lateral inhibition). To assemble a neuron with an elongated and oriented receptive field, as in V1, that neuron must receive inputs from a specific, orderly array of LGN neurons, neurons that have similar receptive fields; in its simplest form, similar LGN neurons would have receptive fields located in a row across the relevant retinal area [76,77]. While it is generally accepted that this model is too simple, something not unlike it must be the case [37]. Thus, to account for our male–female differences, either the properties of the LGN neurons must differ, or the ways in which specific sets of LGN neurons are connected to any given V1 neuron must differ. Having ruled out many of the factors that could account for the sex differences in CSFs, we argue that the differences are most likely due to androgen effects at the cortex. This could be directly at the cortex or could affect projections from LGN to V1 – there are many V1 efferents back to the corresponding LGN regions, perhaps even more than the direct LGN-V1 afferents [78].

### Why sex-differences?

It seems reasonable to conclude that male–female differences in basic sensory capacities are adaptive. Otherwise why is this pattern found across sensory modalities? Specifically, in our case, why is there so heavy an involvement of the sex chromosomes in visual functions?

A plausible reason for sex-differences in spatio-temporal resolution stems from the period when hominids “descended from the trees” and ventured onto the savannas of Africa. Dwellers in forested regions have limited distances over which they must detect and identify objects, be they predators, enemies, or food. On open plains, however, the views are much longer. This places a premium on acuity, for early detection; we reiterate that we find the largest effect sizes at the high spatial frequencies and that our extrapolated acuity data show that males have a 10% advantage in acuity.

The sex-differences in vision might relate to different roles of males and females of early hunter-gatherers; males, being generally larger and more powerful, would have to detect possible predators or prey from afar and also identify and categorize these objects more easily. It is noteworthy that sensitivities to low spatial frequencies is enhanced by temporal modulation; in the real world, retinal images are rarely stationary – objects move and the observer moves.

Evidence for the “hunter-gatherer hypothesis” can be found in studies of visuo-spatial abilities of existing hunter-gatherers: a large meta-analysis of such studies showed that in general males performed better than females [79]. Furthermore, there are significant sex differences in “near-vision” and “far-vision”: males are generally better for accurately perceiving and estimating sizes of targets in far-space [80]. In monkeys there are different populations of cortical neurons dealing with eye-hand coordination and perception of objects in near- or far-space [81]. And this perceptual dichotomy may be related to different neuronal populations associated with the “ventral and dorsal pathways” from primary visual cortex to the higher cortical areas that process visual information. The ventral pathway is from primary visual cortex to infero-temporal cortex and deals with vision-for-identification; the dorsal pathway to the parietal lobe deals more with spatial localization, or vision-for-action [82,83]. Parenthetically, however, we should note that this dorsal/ventral dichotomy is not quite as clear as originally posited (e.g. [84,85]).

Much of the research on sex-effects among hunter-gatherers implicitly assumes that the differences are congenital. However, it has been reported that there is a significant sex-by-age interaction in infants, indicating important maturational factors [86]: females have higher sensitivity at the peak of the CSF at six months, but not at four and eight months – it seems that the sex-related differences we find (overall male superiority) may not be true from birth.

The hunter-gatherer hypothesis correctly predicts that adult males will perform better for targets in far-space – the hunter must perceive and correctly aim at more distant targets – while females will be better for near-space – arguing that they are the gatherers and foragers for nearby foods [87]. Our findings seem to fit this model: males indeed are much more sensitive to high spatial frequencies. However, the sensitivity difference at low spatial frequencies is relatively small and indeed females may do better for static or slowly moving targets (see Figure 2), which would accord with attending to nearby, stationary objects.

## Competing interests

The authors declare that they have no competing interests.

## Authors’ contributions

IA, JG, OF, and AC participated in all aspects of this paper: design, testing participants, data collection and analysis, manuscript preparation. All authors read and approved this final version of the manuscript.
